# Relationships among Local Agricultural Product Purchases, Self-Cooked Meal Consumption, and Healthy Eating Habits: A Cross-Sectional Study in a Town in Gunma, Japan

**DOI:** 10.3390/healthcare10081510

**Published:** 2022-08-11

**Authors:** Daisuke Machida, Yuki Sugiura

**Affiliations:** 1 Home Economics Education Course, Cooperative Faculty of Education, Gunma University, Maebashi 371-8510, Japan; 2 Tamamura Town Health Center, Maebashi 370-1132, Japan

**Keywords:** local agricultural products, cooking, self-cooked meal, breakfast intake, balanced meal intake, vegetable intake, cross-sectional study, Japan

## Abstract

This study examined whether the frequency of purchasing local agricultural products and the intake frequency of self-cooked meals were related to healthy eating habits. A cross-sectional study was conducted using anonymized data from the “Survey on health promotion and food-and-nutritional education” conducted in 2021 in Tamamura, Gunma, Japan, targeting residents aged 20–65. Logistic regression analyses were conducted using the purchasing frequency of local agricultural products (often/sometimes/rarely) and the intake frequency of self-cooked meals (almost every day/not every day) as independent variables. The dependent variables were the frequencies of breakfast (every day/not every day), balanced meal (two times/day or more/fewer than two times/day), and vegetable intake (two times/day or more/fewer than two times/day). The purchasing frequency of local agricultural products was positively related to the frequency of balanced meal and vegetable intake. Additionally, the intake frequency of self-cooked meals was positively related to the frequencies of breakfast, balanced meal, and vegetable intake. In conclusion, significant positive relationships of the purchasing frequency of local agricultural products and the intake frequency of self-cooked meals with healthy eating habits were confirmed.

## 1. Introduction

Healthy eating habits effectively prevent chronic diseases and reduce the risk of death. For example, several meta-analyses reported that breakfast deprivation is a risk factor for obesity, type 2 diabetes, and heart diseases [1,2,3,4,5,6]. In addition, a systematic review of studies of Japanese subjects reported that the intake of a “combined staple meal, main dish, and side dishes,” which is recognized as a well-balanced meal in Japan, is associated with sufficient nutrient intake [7]. Multiple studies also confirmed the association between adherence to the “Japanese Dietary Balance Guide,” which was created by incorporating the concept of staple foods, main dishes, and side dishes, and lower mortality rates [8,9]. Furthermore, many studies revealed that vegetable intake is useful for preventing certain chronic diseases and reducing the associated mortality [10,11,12,13,14,15]. Therefore, it is important to examine the factors that promote healthy eating habits.

In this study, we focused on the purchase of local agricultural products and self-cooking as factors related to the aforementioned healthy eating habits. Many studies have reported positive associations between the purchase of local agricultural products, such as products from farmers’ markets, and fruit and vegetable intake in the United States [16,17,18,19,20]. Similar associations have been also reported in Japan [21,22]. However, the number of studies in Japan is small, and the evidence is limited. Additionally, in recent years, numerous studies have considered the association of cooking at home or eating out with healthy dietary intake in the United Kingdom and United States [23,24,25,26,27]. In Japan, studies have also considered the association of cooking at home or eating out with healthy dietary intake [28,29,30]. However, the results have been inconsistent. For example, regarding the relationship between eating out and balanced meal intake, one study [29] found no significant relationship, but another study [30] recorded a significant inverse relationship. Therefore, further research is needed to clarify these relationships.

The purpose of this study was to examine the associations among the purchasing frequency of local agricultural products, the intake frequency of self-cooked meals, and healthy eating habits among community-dwelling Japanese adults. In Japan, only a few studies have examined the relationships between cooking and eating habits among community-dwelling adults [21,22,28,29,30], and most focus on young adults [28,29,30]. This study fills in the knowledge gaps in the literature by examining the associations between the purchase of local agricultural products, the intake of self-cooked meals, and healthy eating habits among community-dwelling adults in Japan.

## 2. Materials and Methods

### 2.1. Research Design and Survey Overview

A cross-sectional study was conducted using data from the “Survey on health promotion and food-and-nutritional education” conducted in Tamamura, Gunma, Japan [31]. Tamamura is located in the southern part of Gunma, and its population is approximately 36,000 [32]. There are a moderate number of farmers, but this number has been declining in recent years. On the other hand, the amount of land used for commercial and residential purposes is increasing. This survey was conducted to ascertain the residents’ awareness of and interest in health promotion and food and nutrition education, as well as their daily living conditions, and to provide basic data for the interim evaluation of the town’s health promotion and food and nutrition education plan, termed “Hatsuratsu Tamamura 21 (2nd term)” [31]. The survey covered residents aged 20–65 years who lived in Tamamura as of 1 March 2021 [31]. They were randomly sampled from the Basic Resident Ledger, and the selected 1200 participants received self-administered questionnaires by mail [31]. The survey was conducted between April and May 2021 [31]. The number of respondents was 384 (32.0% recovery rate) [31].

The first author received the survey data from the Tamamura Town Health Center. Of the 384 people who provided data, 350 people who had no missing responses regarding the purchasing frequency of local agricultural products, the intake frequency of self-prepared meals, characteristics, and health-related information were used in the final analyses. This study was conducted using only anonymous information on the completed survey, and it was conducted in accordance with the ethical guidelines for life science and medical research involving human subjects in Japan [33].

### 2.2. Variables

#### 2.2.1. Healthy Eating Habit Outcomes

The following indices of healthy eating habits were used: frequency of breakfast intake, frequency of balanced meal intake (a complete set of staple, main, and side dishes), and frequency of vegetable intake. The categories used in the analyses were determined according to the goals of the Basic Plan for the Promotion of Food and Nutritional Education in Japan [34].

The frequency of breakfast intake was assessed using the following question: “Do you eat breakfast?” Responses were categorized using five choices as follows: almost every day, 4–5 days a week, 2–3 days a week, 1 day a week, and almost never. In the Basic Plan for the Promotion of Food and Nutritional Education in Japan, increasing the number of people who eat breakfast every day is set as one of the goals [34]. Therefore, the categories “almost every day” and “not every day (ref.)” were used for the analyses.

The frequency of balanced meal intake was asked using the question “How often do you eat a meal that includes a complete set of staple, main, and side dishes?” Responses were assessed using the following choices: 2 times/day or more, 1 time/day, 4–5 times/week, 2–3 times/week, and less than that or hardly at all. In the Basic Plan for the Promotion of Food and Nutritional Education in Japan, increasing the number of people who eat meals with a complete set of staple, main, and side dishes at least twice a day was set as one of the goals [34]. Therefore, the categories “2 times/day or more” and “less than 2 times/day (ref.)” were used for the analyses.

The frequency of vegetable intake was examined using the question “How often do you eat vegetable dishes?” The question was assessed using five responses as follows: 2 times/day or more, 1 time/day, 4–5 times/week, 2–3 times/week, and less than that or hardly at all. The categories “2 times/day or more” and “less than 2 times/day (ref.)” were used for the analyses. The same categories as those in eating meals with a complete set of staple, main, and side dishes were used.

#### 2.2.2. Independent Variables

The purchasing frequency of local agricultural products was examined using the question “Does your household members purchase agricultural products harvested in Tamamura Town?” The participants responded using three choices: often, sometimes, and not very often (ref.). In the present study, all three responses were used in the analyses as they were.

The intake frequency of self-cooked meals was examined using the question “How often do you eat meals cooked by yourself?” The participants were given four responses as follows: almost every day, a few times a week, a few times a month, and rarely. For this study, the categories “almost every day” and “not every day (ref.)” were used for analyses.

#### 2.2.3. Characteristics and Health-Related Information

The following basic characteristics were used for the analysis: gender (male, female), age (20–39, 40–59, 60–65 years), employment status (employed, unemployed), and family structure (living alone, living together). Additionally, health-related information used for the analyses included body mass index (calculated from self-reported height and weight and classified as less than 18.5, 18.5–24.9, and 25.0 or more), self-rated health (good, not good), smoking status (smoking, smoking in the past, never), drinking status (4 days/week or more, 3 days/week or fewer, never), and exercise habits (with or without exercise habits).

### 2.3. Analyses

First, the chi-squared test was used to confirm the associations among the purchasing frequency of local agricultural products, the intake frequency of self-cooked meals, and each item. Then, binary logistic regression analyses were conducted using the healthy eating habits items as the dependent variables and the purchasing frequency of local agricultural products and intake frequency of self-cooked meals as the independent variables. In the binary logistic regression analyses, odds ratios (OR) and 95% confidence intervals (95% CIs) were calculated using an unadjusted model and a model adjusted for basic characteristics and health-related information. Binary logistic regression analysis was used because the outcome was a two-valued variable. SPSS Statistics 28.0 (IBM Japan, Ltd.) was used for all analyses with a significance level of 5% (two-tailed test).

## 3. Results

### 3.1. Descriptive Statistics

Table 1 presents the distribution of responses to each survey item according to the purchasing frequency of local agricultural products. The chi-squared test revealed significant associations of the purchasing frequency of local agricultural products with gender (*p* < 0.001), age (*p* = 0.009), the frequency of balanced meal intake (*p* < 0.001), and the frequency of vegetable intake (*p* < 0.001).

Table 2 presents the distribution of responses to each survey item according to the intake frequency of self-cooked meals. The chi-squared test revealed significant associations of the intake frequency of self-cooked meals with gender (*p* < 0.001), age (*p* = 0.021), employment status (*p* < 0.001), body mass index (*p* = 0.020), the frequency of breakfast intake (*p* = 0.001), the frequency of balanced meal intake (*p* < 0.001), and the frequency of vegetable intake (*p* = 0.001). A significant association was also found with the purchasing frequency of local agricultural products (*p* < 0.001).

### 3.2. Logistic Regression Analyses

The results of logistic regression analyses are presented in Table 3. There was no significant association between the frequency of breakfast intake and the purchasing frequency of local agricultural products. The frequency of breakfast intake was significantly associated with the intake frequency of self-cooked meals (adjusted model: OR = 2.316, 95% CI = 1.175–4.565, *p* = 0.015).

The frequency of balanced meal intake was significantly associated with both the purchasing frequency of local agricultural products and the intake frequency of self-cooked meals. The adjusted model’s OR for the purchasing frequency of local products was 2.659 (95% CI = 1.250–5.654, *p* = 0.011) for the often purchased category. Regarding the intake frequency of self-cooked meals, the OR was 2.382 (95% CI = 1.318–4.307, *p* = 0.004).

The frequency of vegetable intake was also significantly associated with both the purchasing frequency of local agricultural products and the intake frequency of self-cooked meals. The adjusted model’s OR for the purchasing frequency of local agricultural products was 2.039 (95% CI = 1.153–3.605, *p* = 0.014) and 4.178 (95% CI = 1.932–9.033, *p* < 0.001) for the categories of sometimes and often purchased, respectively. Regarding the intake frequency of self-cooked meals, the OR was 3.383 (95% CI = 1.903–6.014, *p* < 0.001).

## 4. Discussion

This study examined the associations among the purchasing frequency of local agricultural products, the intake frequency of self-cooked meals, and healthy eating habits among community-dwelling adults in Tamamura, Gunma, Japan. The results revealed that the purchasing frequency of local agricultural products had significant positive associations with the frequencies of balanced meal and vegetable intake. The intake frequency of self-cooked meals had significant positive associations with the frequencies of breakfast intake, balanced meal intake, and vegetable intake. This study is one of the few to examine the associations of the purchasing of local agricultural products or intake of self-cooked meals with healthy eating habits among community-dwelling adults in Japan, and it will contribute to future related research.

A significant positive association between the frequencies of breakfast and self-cooked meal intake was confirmed. A previous study of Japanese subjects revealed a positive relationship between the frequencies of cooking and breakfast intake among university students who lived alone [28], which is consistent with the result of this study. In Japan, breakfast is generally consumed at home. Therefore, breakfast is commonly prepared at home. In other words, the association found in this study can be interpreted as a reverse causal relationship in which people often choose to cook breakfast at home as a means of having breakfast.

The frequencies of balanced meal and vegetable intake were positively associated with both the purchasing frequency of local agricultural products and the intake frequency of self-cooked meals. Prior studies have suggested that incentivizing the use of farmers’ markets and shopping at farmers’ markets can increase vegetable intake [16,17,18,19,20,21,22]. In an environment in which local agricultural products are readily available, such as through the presence of farmers’ markets, vegetable intake may increase, which in turn may increase the intake frequency of balanced meals. However, one study found no significant difference in vegetable intake [19], and others observed an increase in vegetable intake but not fruit intake [16,18]. Therefore, these findings should be interpreted cautiously. Additionally, prior studies indicated that cooking at home and not eating out were positively correlated to balanced meal and vegetable intake in the United States and Japan [26,27,30]. Studies of British adults [23] and Japanese pregnant women [29] observed no difference in balanced meal [29] and vegetable intake [23], and people who cooked at home were found to consume less fruit [23]. Although it is true that the present study focusing on Japanese adults found positive associations between cooking with vegetables and balanced meal intake, further research is needed to determine differences by subject, region, and culture. Moreover, there may not be a simple causal relationship through which the purchase of local agricultural products and cooking lead to healthy dietary habits. It is likely that people who want to eat a healthy diet often choose to buy local agricultural products or cook for themselves. Maintaining healthy dietary habits based on takeout food is likely to be burdensome in terms of food access and household finances. This could be explained by the lack of local restaurants with healthy meals, and even if such restaurants are plentiful, they are often expensive. Therefore, people who can cook their own food may find it a less burdensome strategy for maintaining healthy dietary habits. Furthermore, in Tamamura Town, residents can purchase local agricultural products inexpensively in the neighborhood. In addition, many of them grow their own vegetables in home gardens. Thus, the accessibility to locally grown products may be related to the associations observed in this study. This study cannot examine the mechanism of the association among the purchasing frequency of local agricultural products, the intake frequency of self-cooked meals, and healthy eating habits. It will be necessary to elucidate the mechanism of these relationships in the future.

### Limitations

This study had several limitations. First, this study was conducted in a small town in Japan, and generalizing and interpreting the results of this study should be conducted with caution. Second, the response rate of the survey used in this study was low at approximately 32%, and the sample population may not be representative of the entire population. Third, the sample size was small; therefore, stratification analysis was not performed because it would have reduced the statistical power. Fourth, the indicators used in this study were all based on self-reports, and they may not reflect actual behavior because statistical reliability and validity have not been verified. Finally, because this was a cross-sectional study, causal relationships were not confirmed. In the future, large-scale studies using objective information and indicators with confirmed reliability and validity are recommended.

## 5. Conclusions

The purchasing frequency of local agricultural products was positively related to the frequency of balanced meal and vegetable intake. In addition, the intake frequency of self-cooked meals was positively related to the frequencies of breakfast, balanced meal, and vegetable intake. That is, the purchasing frequency of local agricultural products and the intake frequency of self-cooked meals were both significantly and positively associated with healthy eating habits. However, this study had several limitations. In the future, it will be necessary to conduct a longitudinal study with an appropriate sample size and a systematic review and meta-analysis of previous studies.

## Figures and Tables

**Table 1:** Distribution of responses according to the purchasing frequency of local agricultural products (n = 350).

	Purchasing Frequency of Local Agricultural Products						
	Often		Sometimes		Rarely		
	*n*	%	*n*	%	*n*	%	*p*-Value *
	55		176		119		
**Gender**							
Male	22	40.0	65	36.9	71	59.7	<0.001
Female	33	60.0	111	63.1	48	40.3	
**Age**							
20–39	13	23.6	42	23.9	50	42.0	0.009
40–59	27	49.1	97	55.1	47	39.5	
60–65	15	27.3	37	21.0	22	18.5	
**Employment**							
Unemployed (including housewives and students)	12	21.8	34	19.3	16	13.4	0.296
Employed	43	78.2	142	80.7	103	86.6	
**Family structure**							
Living alone	2	3.6	13	7.4	13	10.9	0.235
Living together	53	96.4	163	92.6	106	89.1	
**Self-rated health**							
Not good	5	9.1	16	9.1	21	17.6	0.066
Good	50	90.9	160	90.9	98	82.4	
**Body mass index**							
18.5–24.9	42	76.4	132	75.0	74	62.2	0.091
Less than 18.5	2	3.6	11	6.3	7	5.9	
25.0 or more	11	20.0	33	18.8	38	31.9	
**Smoking status**							
Never	31	56.4	99	56.3	64	53.8	0.457
Former smoking	13	23.6	46	26.1	40	33.6	
Current smoking	11	20.0	31	17.6	15	12.6	
**Drinking status**							
Never	19	34.5	93	52.8	65	54.6	0.067
3 days/week or fewer	15	27.3	45	25.6	28	23.5	
4 days/week or more	21	38.2	38	21.6	26	21.8	
**Exercise habits**							
No	37	67.3	116	65.9	85	71.4	0.604
Yes	18	32.7	60	34.1	34	28.6	
**Breakfast intake**							
Not every day	8	14.5	35	19.9	31	26.1	0.197
Almost every day	47	85.5	139	79.0	88	73.9	
Missing	0	0.0	2	1.1	0	0.0	
**Balanced meal intake**							
Fewer than 2/day	22	40.0	97	55.1	84	70.6	<0.001
2/day or more	33	60.0	79	44.9	35	29.4	
**Vegetable intake**							
Fewer than 2/day	18	32.7	92	52.3	87	73.1	<0.001
2/day or more	37	67.3	84	47.7	32	26.9	

* Chi-squared test.

**Table 2:** Distribution of responses according to the intake frequency of self-cooked meals (*n* = 350).

	Intake Frequency of Self-Cooked Meals				
	Almost Every Day		Not Every Day		
	*n*	%	*n*	%	*p*-Value *
	188		162		
**Gender**					
Male	39	20.7	119	73.5	<0.001
Female	149	79.3	43	26.5	
**Age**					
20–39	45	23.9	60	37.0	0.021
40–59	97	51.6	74	45.7	
60–65	46	24.5	28	17.3	
**Employment**					
Unemployed (including housewives and students)	46	24.5	16	9.9	<0.001
Employed	142	75.5	146	90.1	
**Family structure**					
Living alone	14	7.4	14	8.6	0.681
Living together	174	92.6	148	91.4	
**Self-rated health**					
Not good	23	12.2	19	11.7	0.885
Good	165	87.8	143	88.3	
**Body mass index**					
18.5–24.9	140	74.5	108	66.7	0.020
Less than 18.5	14	7.4	6	3.7	
25.0 or more	34	18.1	48	29.6	
**Smoking status**					
Never	112	59.6	82	50.6	0.243
Former smoking	48	25.5	51	31.5	
Current smoking	28	14.9	29	17.9	
**Drinking status**					
Never	102	54.3	75	46.3	0.071
3 days/week or fewer	38	20.2	50	30.9	
4 days/week or more	48	25.5	37	22.8	
**Exercise habits**					
No	123	65.4	115	71.0	0.266
Yes	65	34.6	47	29.0	
**Breakfast intake**					
Not every day	27	14.4	47	29.0	0.001
Almost every day	159	84.6	115	71.0	
Missing	2	1.1		0.0	
**Balanced meal intake**					
Fewer than 2/day	89	47.3	114	70.4	<0.001
2/day or more	99	52.7	48	29.6	
**Vegetable intake**					
Fewer than 2/day	74	39.4	123	75.9	<0.001
2/day or more	114	60.6	39	24.1	
**Purchasing frequency of local agricultural products**					
Often	39	20.7	16	9.9	<0.001
Sometimes	104	55.3	72	44.4	
Rarely	45	23.9	74	45.7	

* Chi-squared test.

**Table 3:** Purchasing frequency of local agricultural products, intake frequency of self-cooked meals, and healthy eating habits (logistic regression models).

	Crude Model		Adjusted Model	
*Dependent variables*	OR (95% CI)	*p*-Value	OR (95% CI)	*p*-Value
***Breakfast intake***				
**Purchasing frequency of local agricultural products**				
Rarely	1 (Reference)		1 (Reference)	
Sometimes	1.399 (0.805–2.430)	0.233	1.049 (0.556–1.980)	0.881
Often	2.069 (0.880–4.862)	0.095	1.542 (0.582–4.090)	0.383
**Intake frequency of self-cooked meals**				
Not every day	1 (Reference)		1 (Reference)	
Almost every day	2.406 (1.415–4.091)	0.001	2.316 (1.175–4.565)	0.015
***Balanced meal intake***				
**Purchasing frequency of local agricultural products**				
Rarely	1 (Reference)		1 (Reference)	
Sometimes	1.954 (1.193–3.202)	0.008	1.610 (0.919–2.821)	0.095
Often	3.599 (1.845–7.022)	<0.001	2.659 (1.250–5.654)	0.011
**Intake frequency of self-cooked meals**				
Not every day	1 (Reference)		1 (Reference)	
Almost every day	2.641 (1.697–4.111)	<0.001	2.382 (1.318–4.307)	0.004
***Vegetable intake***				
**Purchasing frequency of local agricultural products**				
Rarely	1 (Reference)		1 (Reference)	
Sometimes	2.482 (1.503–4.099)	<0.001	2.039 (1.153–3.605)	0.014
Often	5.588 (2.792–11.18)	<0.001	4.178 (1.932–9.033)	<0.001
**Intake frequency of self-cooked meals**				
Not every day	1 (Reference)		1 (Reference)	
Almost every day	4.858 (3.054–7.727)	<0.001	3.383 (1.903–6.014)	<0.001

OR, odds ratio; CI, confidence interval. Dependent variables: breakfast intake, balanced meal intake, and vegetable intake. Adjusted models: adjusted for gender, age, employment status, family structure, body mass index, self-rated health, smoking status, drinking status, and exercise habits. The intake frequency of self-cooked meals and purchasing frequency of local agricultural products were included together in the adjusted models.

## Data Availability

The raw data are not publicly available due to ethical restrictions.
